# The alleviating effects and mechanisms of *Enterococcus faecium* Kimate-X and *Lactobacillus plantarum* Kimate-F combination on canine inflammatory bowel disease

**DOI:** 10.3389/fvets.2025.1534665

**Published:** 2025-05-06

**Authors:** Rui Zhang, Wanjin Hu, Saiwei Zhong, Weiyang Chen, Shuang Xie, Meiru Chen, Qinghua Yu

**Affiliations:** ^1^College of Veterinary Medicine, Nanjing Agricultural University, Nanjing, China; ^2^Laboratory of Microbiology, Immunology and Metabolism, Diprobio (Shanghai) Co., Limited, Shanghai, China; ^3^College of Animal Science and Technology of Jiangxi Agricultural University, Nanchang, China

**Keywords:** canine inflammatory bowel disease, probiotics, *Enterococcus faecium*, *Lactobacillus plantarum*, dysbiosis, inflammation

## Abstract

**Introduction:**

Canine inflammatory bowel disease (IBD) is characterized by chronic intestinal inflammation and dysbiosis. Conventional treatments often result in adverse effects and contribute to antibiotic resistance, highlighting the need for safe, effective alternatives. Probiotics have gained attention for their potential in modulating gut microbiota and immune responses. This study investigates the therapeutic mechanisms of *Enterococcus faecium* Kimate-X and *Lactobacillus plantarum* Kimate-F, individually and in combination, in alleviating canine IBD.

**Methods:**

*In vitro* antibacterial and anti-inflammatory activities were assessed using agar well diffusion assays and LPS-induced RAW 264.7 macrophages, respectively. *In vivo* efficacy was evaluated in dextran sulfate sodium (DSS)-induced colitis models in mice and dogs. Metagenomic sequencing was performed on canine fecal samples to analyze microbiota composition and functional pathways. Short-chain fatty acid (SCFA) levels were quantified, and key host signaling pathways were examined.

**Results:**

Kimate-F showed strong antibacterial effects against *Escherichia coli*, *Salmonella enteritidis*, and *Yersinia enterocolitica*. Kimate-X significantly suppressed nitric oxide (NO) and TNF-α production in the *in vitro* inflammation model. In both mouse and canine DSS-induced colitis models, the probiotic combination significantly reduced weight loss, colonic damage, and serum inflammatory cytokines, while increasing IL-10 levels. Metagenomic analysis revealed enhanced microbial diversity, with enrichment of *Bifidobacterium* species and upregulation of metabolic pathways involved in nutrient absorption and immune regulation. The probiotic combination also modulated the PPAR and AMPK signaling pathways and promoted SCFA production in canine feces.

**Discussion:**

These findings suggest that *E. faecium* Kimate-X and *L. plantarum* Kimate-F act synergistically to restore gut homeostasis, reduce intestinal inflammation, and enhance host immunity. Their ability to modulate gut microbiota composition, host signaling, and metabolic output underscores their potential as safe and effective probiotic candidates for managing canine IBD.

## Introduction

Dogs are among the earliest domesticated animals and have played a significant role in human society for thousands of years (1). With economic development and rising living standards, the number of pet dogs has significantly increased (2, 3). It is estimated that the global population of pet dogs has reached between 700 million and 1 billion, and this number continues to grow (4). In addition to providing emotional support and companionship, dogs play crucial roles in various fields such as guide work, drug detection, and search and rescue operations (5, 6). Consequently, canine health has become a growing concern, particularly gastrointestinal disorders, which significantly impact dogs’ well-being and quality of life (7).

IBD is a chronic and debilitating gastrointestinal disorder in dogs, characterized by chronic inflammation and histopathological changes in the intestinal mucosa with an unclear etiology (8, 9). The clinical symptoms of canine IBD include diarrhea, vomiting, reduced appetite, and weight loss, which severely impair the affected dogs’ health (10). Although the exact cause of canine IBD remains unclear, it is widely believed to be associated with multiple factors, including genetics, immune-mediated mechanisms, environmental factors, and gut microbiota dysbiosis (11, 12).

IBD is closely linked to gut dysbiosis, particularly characterized by the overgrowth of harmful bacteria such as *Escherichia coli* and *Salmonella* (13, 14). Studies have demonstrated that dogs with IBD exhibit significantly reduced gut microbial diversity and an increased relative abundance of opportunistic pathogens (15, 16). The proliferation of these harmful bacteria may exacerbate intestinal inflammation, damage the mucosal barrier, and further perpetuate the inflammatory cycle (17). Gut dysbiosis is now recognized as a key factor in the onset and progression of IBD, making the modulation of gut microbiota a promising therapeutic strategy (18).

Currently, traditional treatments for canine IBD mainly rely on immunosuppressants (e.g., corticosteroids) and antibiotics (19). However, the long-term use of these drugs can lead to various side effects, such as immune suppression, increased risk of infection, and the development of drug resistance, which limit their prolonged use (20, 21). As a result, there is increasing interest in identifying safe and effective alternative therapies.

Probiotics, as natural and well-tolerated biological agents, have been widely used to promote gut health in both humans and animals (22, 23). Probiotics are defined as “live microorganisms that, when administered in adequate amounts, confer a health benefit on the host” (24). Their mechanisms of action include inhibiting the colonization and growth of pathogenic microorganisms, enhancing intestinal barrier function, modulating the immune system, and producing beneficial metabolites, such as SCFAss (25, 26). In studies involving dogs, probiotics have been shown to improve gut microbial composition, alleviate gastrointestinal symptoms, and enhance immune function (27, 28). Particularly in the treatment of IBD, probiotics have demonstrated potential therapeutic effects by regulating immune responses and restoring microbial balance in the gut (29, 30).

Dextran sulfate sodium (DSS) is a widely used chemical reagent that induces colitis in animals by damaging the intestinal epithelial barrier, and it is frequently used in IBD animal model research (31, 32). The DSS-induced colitis model shares similar pathological features with canine IBD, such as mucosal damage, inflammatory cell infiltration, and the upregulation of pro-inflammatory cytokine expression (33, 34). However, studies on DSS-induced colitis models in dogs are relatively limited, with most research focusing on rodent models. This constraint limits our understanding of the pathophysiological mechanisms and treatment strategies for canine IBD.

*Enterococcus faecium* and *Lactobacillus plantarum* are two commonly studied probiotic strains that have demonstrated various biological functions, including antimicrobial, anti-inflammatory, and immunomodulatory activities (35, 36). *E. faecium* produces antimicrobial substances such as lactic acid, hydrogen peroxide, and bacteriocins, which inhibit the growth of pathogenic bacteria (37). Its cell wall components can also modulate the host’s immune response by promoting the production of anti-inflammatory cytokines (38). *L. plantarum* is well-adapted to the gastrointestinal tract, where it adheres to intestinal epithelial cells and strengthens barrier function (39). It also inhibits pathogens through the production of bacteriocins and organic acids (40). Studies have suggested that probiotic combinations may exert synergistic effects, enhancing their anti-inflammatory and immunomodulatory functions (41).

This study aims to elucidate the potential benefits of probiotics in managing IBD in dogs. Specifically, it evaluates the efficacy of *Enterococcus faecium* Kimate-X and *Lactobacillus plantarum* Kimate-F in alleviating DSS-induced colitis, investigates whether their combination exerts synergistic effects enhancing anti-inflammatory and immunomodulatory properties, and employs metagenomic sequencing and metabolomic analysis to assess the impact of these probiotics on gut microbiota composition and metabolic pathways. Based on these objectives, we hypothesize that the combined use of these probiotics will produce more pronounced anti-inflammatory effects and improved gut microbial balance, thereby offering a promising approach for canine IBD prevention and management and providing valuable insights for understanding human IBD.

## Materials and methods

### Antibacterial activity of Kimate-X and Kimate-F against intestinal pathogens

The agar diffusion method was used to evaluate the antibacterial effects of *Enterococcus faecium* Kimate-X and *Lactobacillus plantarum* Kimate-F against common pathogens associated with canine enteritis. The pathogens selected for this experiment included *Enterotoxigenic Escherichia coli* ATCC 43888, *Salmonella enterica* subsp. *enterica* serovar Typhimurium ATCC 14028, and *Yersinia enterocolitica* ATCC 9610. Each pathogen was cultured in suitable media until reaching the logarithmic growth phase and then diluted to a concentration of 1 × 10^8^ CFU/mL. One milliliter of each pathogen suspension was spread evenly onto LB agar plates, and after standing for 1 min, any excess liquid was removed. Three wells were made in each plate using an 8-mm sterile punch. The first well was filled with 50 μL of *Lactobacillus plantarum* Kimate-F suspension (1 × 10^8^ CFU/mL), the second well was filled with the same concentration of *Enterococcus faecium* Kimate-X suspension, and the third well, serving as a control, was filled with 50 μL of sterile saline. Each condition was tested in triplicate. Plates were incubated anaerobically at 37°C for 24 h, and inhibition zones were measured in millimeters using calipers to quantify the inhibitory effects of Kimate-X and Kimate-F on each pathogen.

### Immunomodulatory effects of Kimate-X and Kimate-F in an LPS-induced inflammation model

RAW 264.7 macrophages were cultured in Dulbecco’s Modified Eagle’s Medium (DMEM) supplemented with 10% fetal bovine serum (FBS) and 1% antibiotics. The cells were incubated at 37°C in a 5% CO₂ incubator. When cells reached 80–90% confluence, they were digested with 0.25% trypsin-EDTA solution and resuspended at 5 × 10^5^ cells/mL. Next, 100 μL of this suspension (approximately 5 × 10^4^ cells) was seeded into each well of a 96-well plate and incubated for 24 h to allow cell adhesion. Inflammation was induced by treating the macrophages with 0.5 μg/mL lipopolysaccharide (LPS). Six hours after LPS stimulation, 1 × 10^5^ CFU/mL of Kimate-X and Kimate-F were added to the respective treatment groups. After 12 h of incubation, supernatants were collected, and nitric oxide (NO) levels were measured using a commercial ELISA kit (Meilian Bio, Shanghai). NO levels in each group were compared to evaluate the effects of the probiotic suspensions on LPS-induced NO production.

For the evaluation of LPS-induced TNF-α production, the same cell culture methods and groupings were employed. RAW 264.7 cells were treated with 500 ng/mL LPS to induce inflammation, and 1 × 10^5^ CFU/mL of Kimate-X or Kimate-F was added to the treatment groups. The control group received sterile saline without LPS or probiotics, and the inflammation model group received only LPS. After 12 h of incubation, supernatants were collected and stored at −80°C. TNF-α levels were measured using a commercial ELISA kit (Meilian Bio, Shanghai) to assess the regulatory effects of probiotics on LPS-induced TNF-α production.

### Mouse experiment design

A DSS-induced colitis model was used to evaluate the effects of Kimate-X, Kimate-F, and their combination on colitis in mice. Thirty 5-week-old male C57BL/6 mice were randomly assigned to five groups (*n* = 6 per group): Control, DSS model, Kimate-X, Kimate-F, and mixed probiotics (Kimate-X + Kimate-F). The Kimate-X group received *Enterococcus faecium* Kimate-X, the Kimate-F group received *Lactobacillus plantarum* Kimate-F, and the mixed probiotics group received a 1:1 combination of both strains. The Control group received PBS. Mice were administered 200 μL of the appropriate suspension (1 × 10^9^ CFU/mL) daily by oral gavage. After 7 days, all groups except the Control were given 3% DSS (molecular weight 36–50 kDa) in their drinking water to induce colitis. During the experiment, body weight, stool consistency, and bleeding were recorded daily to calculate the disease activity index (DAI). Samples were collected once the DSS model group exhibited clear colitis symptoms.

### Sample collection and analysis

Blood samples were collected from the orbital venous plexus, and serum was separated by centrifugation at 3,000 rpm for 10 min after standing at room temperature for 30 min. Serum levels of LPS, IL-10, and TNF-α were measured using commercial ELISA kits (Meilian Bio, Shanghai). After euthanasia, spleens were collected to calculate the spleen index, and colon tissues were harvested for histological analysis, including colon length measurement and inflammation scoring.

### Canine experiment design

The experimental design is shown in Figure 3A. Sixteen 5-month-old male Beagle dogs were randomly divided into two groups (*n* = 8 per group): Control and probiotic-treated (Kimate-X + Kimate-F). The Control group received a basic diet, while the probiotic-treated group received a basic diet plus a daily oral administration of mixed Kimate-X and Kimate-F suspension (1 × 10^9^ CFU/day for each strain) for the first 7 days. From days 8 to 14, the Control group was given 4% DSS (molecular weight 36–50 kDa) solution to induce colitis, while the probiotic-treated group received both probiotics and DSS. Body weight was recorded every 2 days, and the DAI was calculated. Blood and fecal samples, were collected on day 14. The mental state and fecal scoring were assessed according to the method described by Nestler et al. (42).

### Canine blood and fecal sample collection and analysis

On day 14, 5 mL of blood was collected from the forelimb veins of each dog and centrifuged to obtain serum, which was stored at −80°C for subsequent analysis. Serum inflammatory cytokines (IL-1β, TNF-α, LPS, and IL-10) were measured using commercial ELISA kits (Meilian Bio, Shanghai). Fresh fecal samples were collected, immediately frozen at −80°C, and analyzed for SCFAs. All experimental procedures were approved by the Animal Ethics Committee of Nanjing Agricultural University (Approval Number: NJAU. NO20231227193).

### Canine fecal short-chain fatty acid analysis

SCFAs analysis was performed at Shanghai Zhongke New Life Biotechnology Co., Ltd. by gas chromatography-mass spectrometry (GC-MS). Fecal samples were thawed, resuspended in 20% phosphoric acid, and processed for GC-MS analysis, with methylvaleric acid as the internal standard. Samples were analyzed using an Agilent DB-FFAP capillary column, and SCFAs concentrations were quantified based on calibration curves.

### DNA extraction and shotgun metagenomic sequencing

Genomic DNA was extracted from fecal samples using the QIAamp PowerFecal Pro DNA Kit (QIAGEN, United States). DNA quality was assessed by agarose gel electrophoresis and quantified using a Qubit Fluorometer. Libraries were prepared using the NEBNext DNA Library Prep Kit, and sequencing was performed on an Illumina Novaseq 6000 platform. Raw reads were filtered, host DNA was removed, and non-host reads were assembled for gene prediction.

### Microbial taxonomy and functional profiling

Taxonomic profiling was performed using MetaPhlAn4, providing species-level resolution of gut microbial composition, while functional annotations were carried out using EggNOG mapper to classify genes into metabolic pathways and biological functions. Alpha diversity indices (Shannon index, Inverse Simpson index) were calculated to assess microbial richness and evenness, while beta diversity was evaluated using Bray–Curtis dissimilarity and principal coordinate analysis (PCoA) to compare microbiota compositions across experimental groups. Group differences in microbial diversity and composition were assessed using Wilcoxon rank-sum tests for pairwise comparisons and PERMANOVA (permutational multivariate analysis of variance) for overall community structure variations. Functional pathway enrichment analysis was conducted using KEGG (Kyoto Encyclopedia of Genes and Genomes) and LEfSe (Linear Discriminant Analysis Effect Size) analysis to identify differentially enriched metabolic pathways between groups.

### Correlation analysis

Spearman correlation analysis was conducted to examine associations between gut microbiota, functional gene pathways, SCFAs, and inflammatory markers. False discovery rate (FDR) correction was applied to control for multiple testing errors, with adjusted *p*-values <0.05 considered statistically significant. Correlation heatmaps were generated to visualize key relationships, while network analysis using Cytoscape identified highly correlated features (*r* > 0.5 or *r* < −0.5, *p* < 0.05), mapping microbial taxa, functional genes, and immune responses in an integrated network.

### Statistical analysis

All statistical analyses were conducted using SPSS 26.0, and figures were generated using GraphPad Prism 8.0. Group comparisons were made using Student’s t-test, with statistical significance set at *p* < 0.05.

## Results

### *In vitro* results

**Figure 1:**
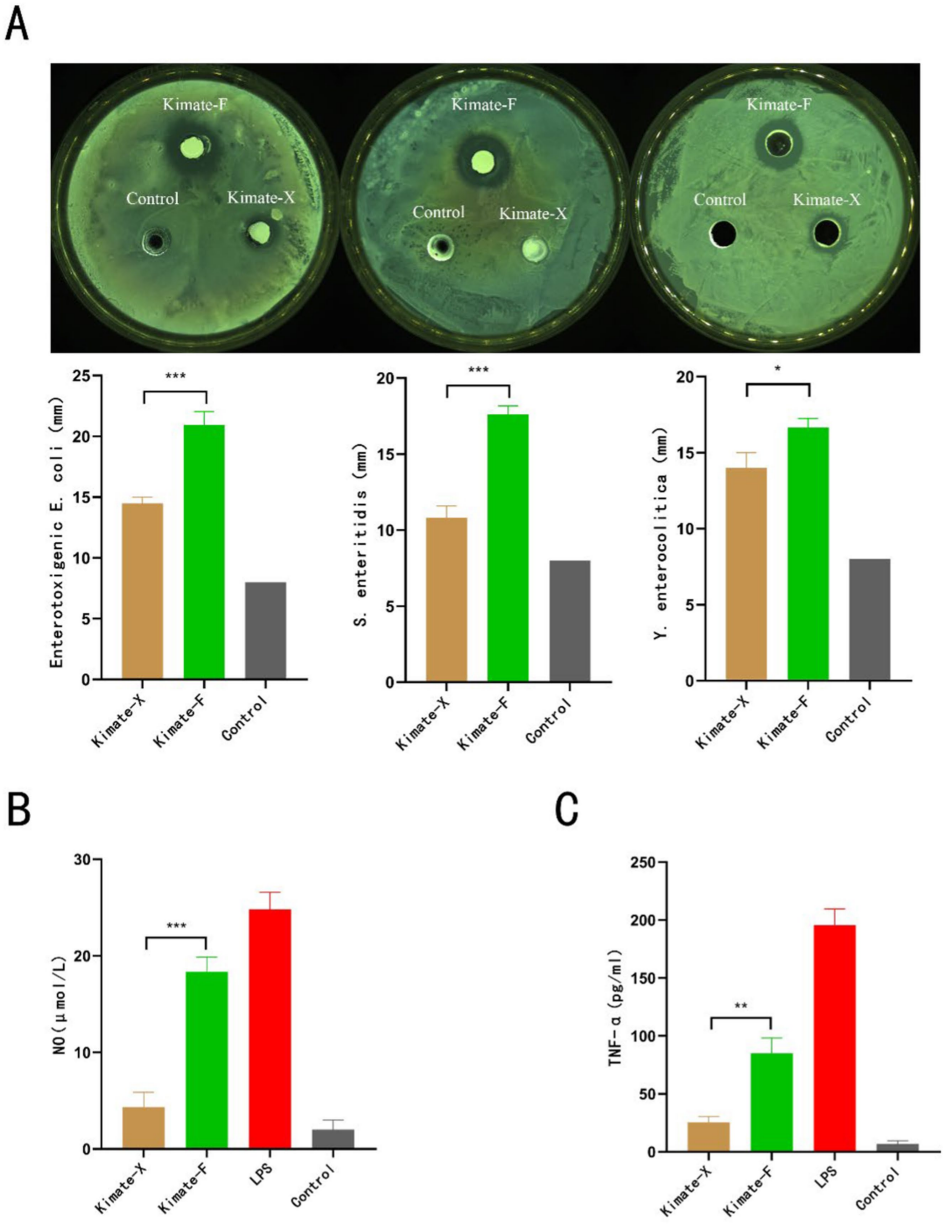
Antimicrobial and immunomodulatory effects of probiotic strains Kimate-X and Kimate-F. **(A)** Inhibitory effects of Kimate-X and Kimate-F against Enterotoxigenic *Escherichia coli*, *Salmonella enteritidis*, and *Yersinia enterocolitica*, as evaluated by agar well diffusion assay. Representative images are shown (top), and inhibition zone diameters are quantified (bottom). **(B)** Effects on NO production: Kimate-X and Kimate-F on NO levels in LPS-induced RAW 264.7 cells. **(C)** TNF-α expression: effects of Kimate-X and Kimate-F on TNF-α expression in LPS-induced RAW 264.7 cells. Data are presented as mean ± SEM, statistical significance is indicated by *P < 0.05, **P < 0.01, ***P < 0.001.

The antibacterial activity of *Enterococcus faecium* Kimate-X and *Lactobacillus plantarum* Kimate-F was evaluated using the agar diffusion method. As shown in (Figure 1A), both probiotic strains inhibited the growth of *Escherichia coli*, *Salmonella enteritidis*, and *Yersinia enterocolitica*, while no inhibition zones were observed in the control group. For *E. coli*, the inhibition zone diameter of Kimate-F (21 ± 1 mm) was significantly larger than that of Kimate-X (14.5 ± 0.5 mm, *P* < 0.001), indicating a stronger antibacterial effect of Kimate-F. Similar results were observed for *S. enteritidis*, with Kimate-F exhibiting a significantly greater inhibition zone (17.6 ± 0.6 mm) than Kimate-X (10.5 ± 0.5 mm, *P* < 0.001). Against *Y. enterocolitica*, Kimate-F again demonstrated superior antibacterial activity (17.5 ± 0.46 mm) compared to Kimate-X (14.3 ± 0.52 mm, *P* < 0.05).

In the LPS-induced RAW 264.7 macrophage inflammation model, both Kimate-X and Kimate-F significantly reduced NO and TNF-α levels. The NO levels in the Kimate-X group were significantly lower than those in the Kimate-F group (*p* < 0.001) (Figure 1B). In terms of TNF-α levels (Figure 1C), the Kimate-X group exhibited significantly lower levels compared to the Kimate-F group (*p* < 0.01).

### Mouse experiment results

**Figure 2:**
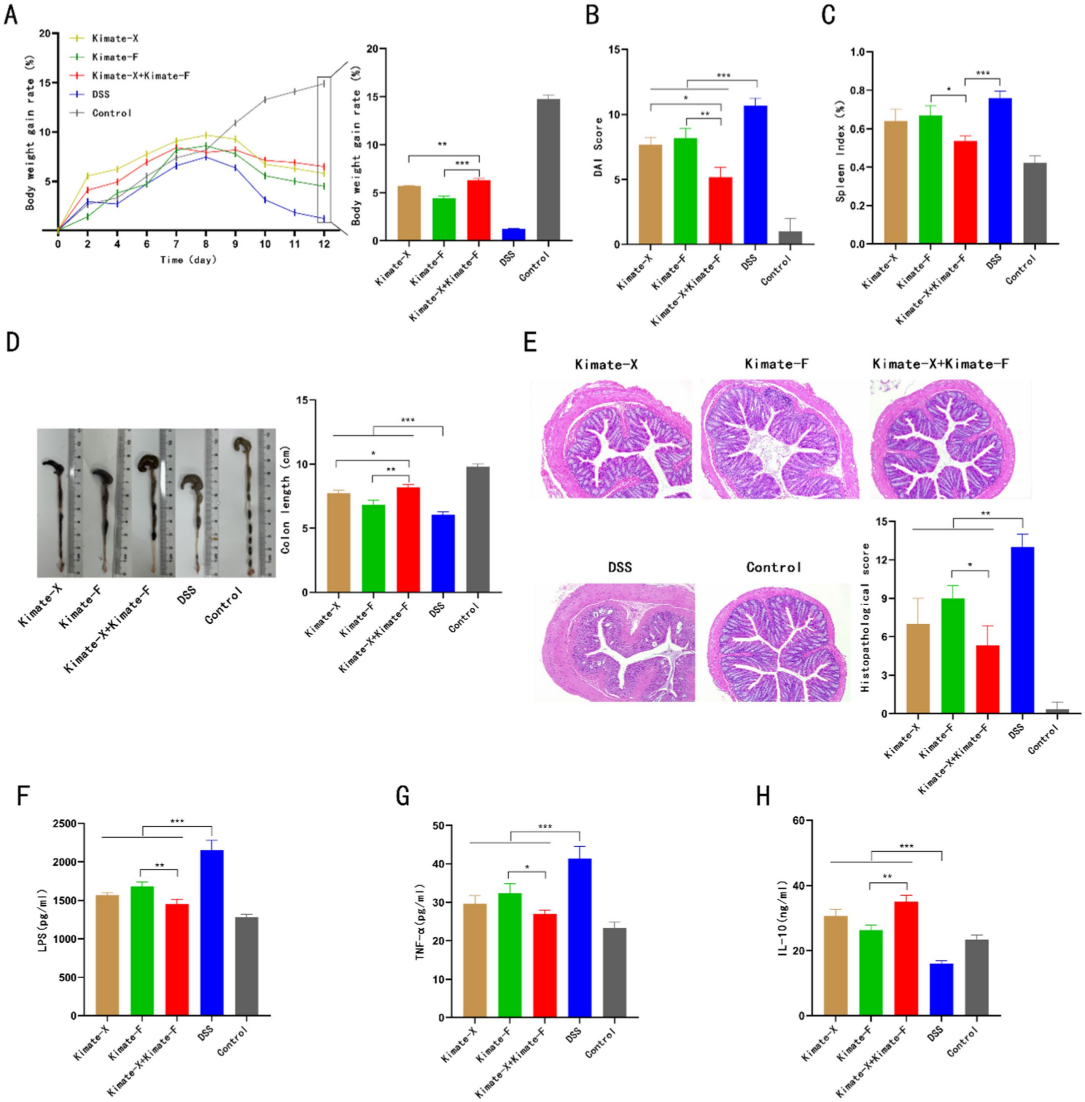
Protective effects of Kimate-X, Kimate-F, and their combination in a DSS-induced colitis mouse model. **(A)** Body weight changes: left panel shows body weight changes under DSS induction; right panel displays statistical analysis of body weight gain rates on day 12 for each group. **(B)** DAI scores on day 12 for each group. **(C)** Spleen index (SI) for each group. **(D)** Colon length comparison among groups. **(E)** Histopathological scores: left panel shows H&E staining of colon tissue, highlighting structural damage in the DSS group and protection by Kimate-F and the combination group. Right panel presents histopathological score results. **(F–H)** Serum levels of LPS **(F)**, TNF-α **(G)**, and IL-10 **(H)** across groups. Data are presented as mean ± SEM, statistical significance is indicated by *p < 0.05, **p < 0.01, and ***p < 0.001.

In the DSS-induced colitis mouse model, the effects of Kimate-X, Kimate-F, and their combination on body weight, disease activity index (DAI), spleen index, colon length, histopathological changes, and inflammatory markers were evaluated. Regarding body weight changes (Figure 2A), the DSS group showed significant weight loss, whereas the control group experienced continuous weight gain. Kimate-X, Kimate-F, and the mixed probiotic group significantly alleviated weight loss, with the mixed probiotic group demonstrating the greatest weight maintenance (*p* < 0.001). In the DAI (Figure 2B), the DSS group exhibited significantly higher scores, while Kimate-X, Kimate-F, and the mixed probiotic group significantly reduced DAI scores, with the mixed probiotic group showing the most pronounced improvement (*p* < 0.05).

For the spleen index (Figure 2C), the DSS group exhibited the highest index, and Kimate-X, Kimate-F, and the mixed probiotic groups significantly reduced spleen indices, with the mixed probiotic group showing the greatest reduction (*p* < 0.05). In terms of colon length (Figure 2D), the DSS group exhibited significantly shortened colons, while Kimate-X, Kimate-F, and the mixed probiotic groups alleviated this shortening, with the mixed probiotic group showing the longest colon length, significantly superior to the single probiotic groups (*p* < 0.001).

In histopathological scoring (Figure 2E), the DSS group exhibited the most severe pathological damage, with significantly higher scores than other groups. Kimate-X, Kimate-F, and the mixed probiotic groups significantly reduced histopathological scores, with the mixed probiotic group showing markedly better tissue repair than the single probiotic groups (*p* < 0.01). Inflammatory marker analysis (Figures 2F–H) revealed that the DSS group exhibited significantly elevated serum LPS and TNF-α levels, while IL-10 levels were significantly reduced. In contrast, Kimate-X, Kimate-F, and the mixed probiotic groups significantly lowered LPS and TNF-α levels and increased IL-10 levels, with the mixed probiotic group demonstrating superior results compared to the single probiotic groups (*p* < 0.001).

### Canine experiment results

**Figure 3:**
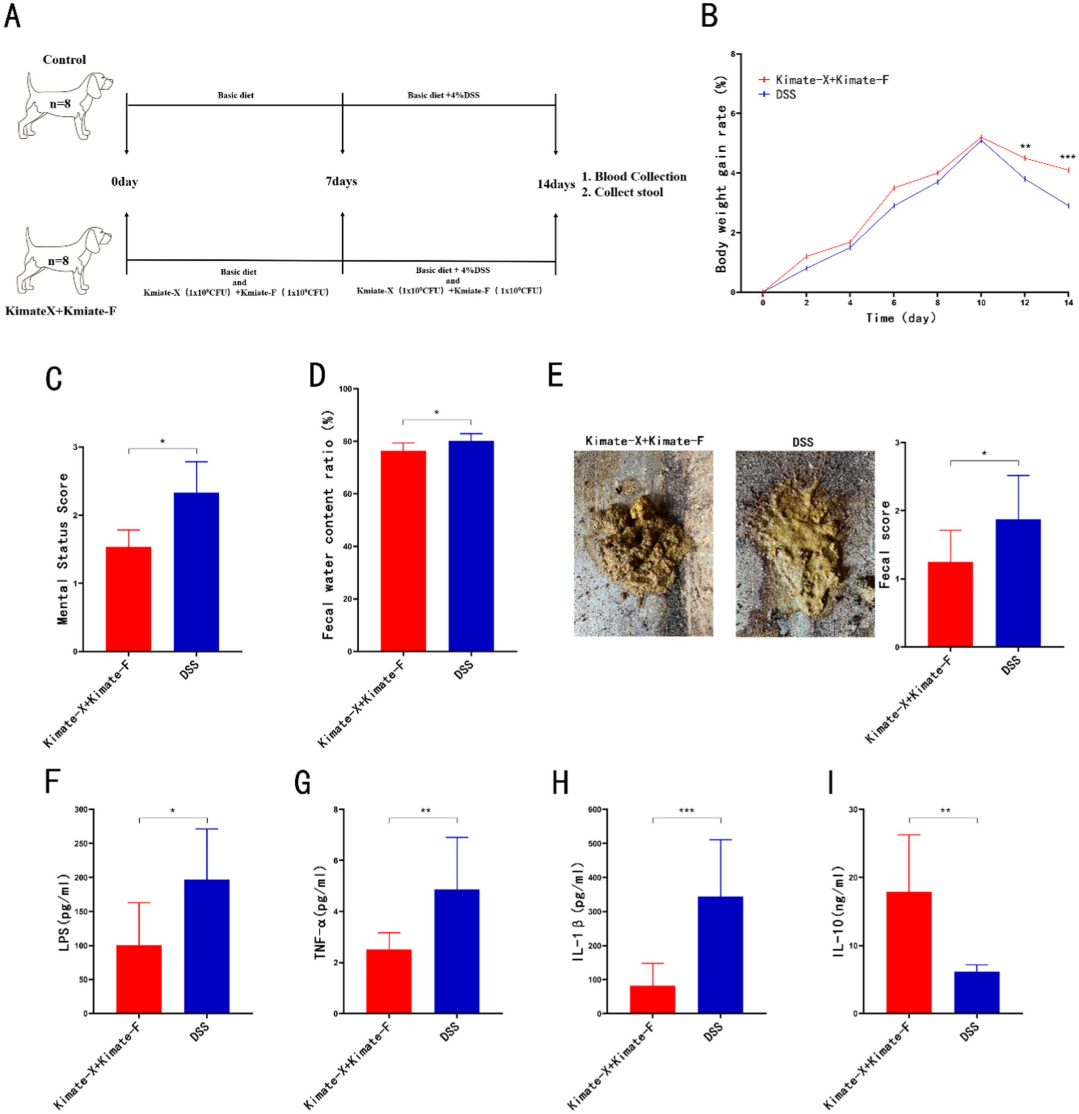
Protective effects of Kimate-X and Kimate-F combination in a DSS-induced canine colitis model. **(A)** Experimental protocol schematic for the canine study. **(B)** Body weight change curves for the two groups. **(C)** Mental state scores of dogs on day 14. **(D)** Fecal water content on day 14. **(E)** Fecal appearance and scores on day 14. **(F–I)** Serum levels of LPS **(F)**, TNF-α **(G)**, IL-1β **(H)** and IL-10 **(I)** on day 14. Data are presented as mean ± SEM, statistical significance is indicated by *p < 0.05, **p < 0.01, and ***p < 0.001.

In the DSS-induced colitis experiment in dogs (Figure 3A), the weight gain in the mixed probiotic group was significantly higher than in the DSS group (Figure 3B, *p* < 0.01), while mental status scores were significantly lower in the mixed probiotic group compared to the DSS group (Figure 3C, *p* < 0.05). Fecal analysis showed that the water content and fecal scores in the mixed probiotic group were significantly lower than those in the DSS group (Figures 3D,E, *p* < 0.05). Serum inflammatory markers revealed that LPS, TNF-α, and IL-1β levels in the mixed probiotic group were significantly lower than in the DSS group (Figures 3F–H, *p* < 0.05, *p* < 0.01, and *p* < 0.001, respectively). The level of the anti-inflammatory immune marker IL-10 in the mixed probiotic group was significantly higher than that in the DSS group (Figure 3I, *P* < 0.01).

### Canine gut microbiota analysis

**Figure 4:**
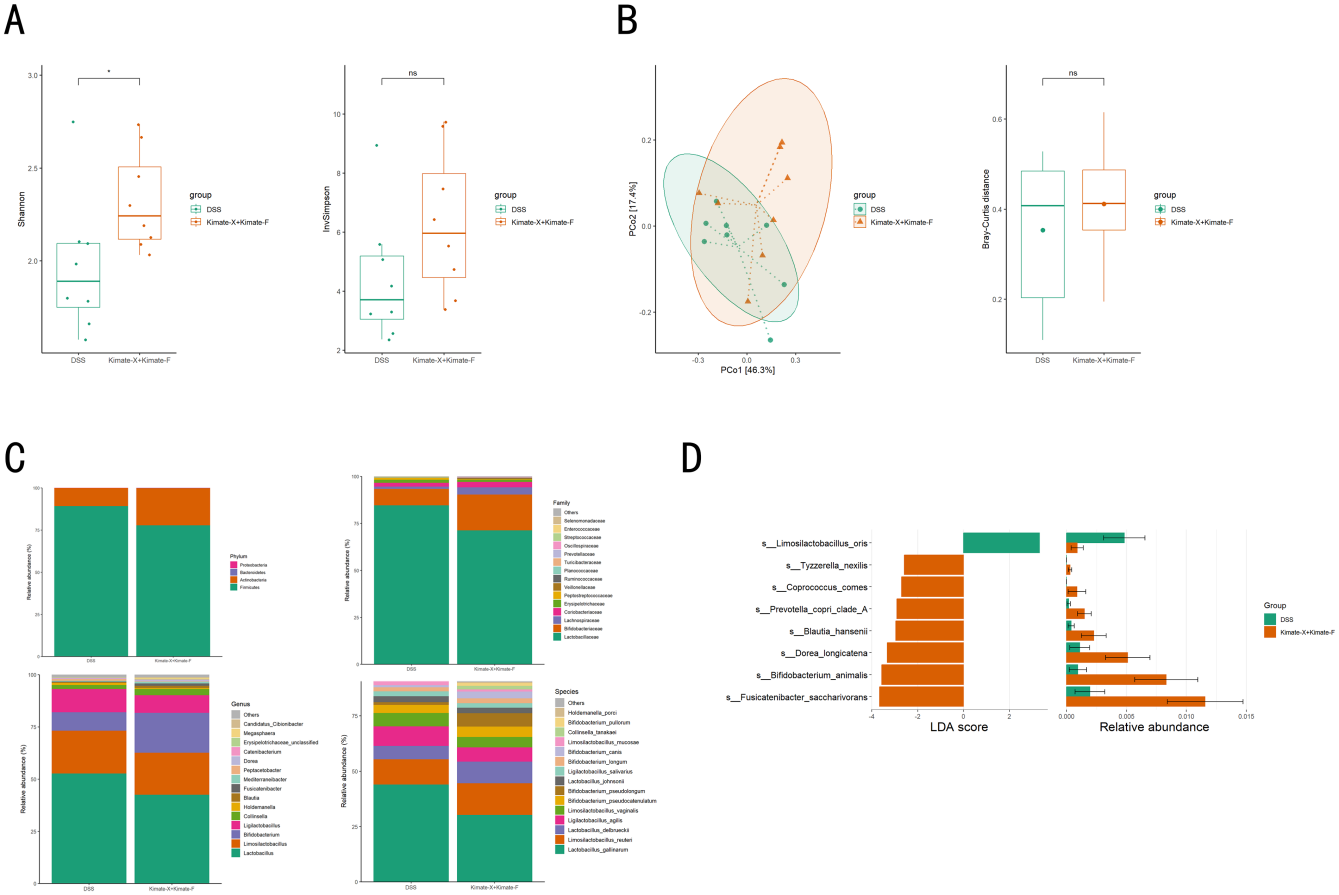
Effects of Kimate-X and Kimate-F combination on gut microbiota in DSS-induced colitis dogs. **(A)** Gut microbiota diversity: Shannon index (left) and Invsimpson index (right). **(B)** Microbiota community structure: principal coordinate analysis (PCoA, left) and Bray–Curtis distance index (right). **(C)** Microbial composition: relative abundances at the phylum (top left), family (top right), genus (bottom left), and species (bottom right) levels. **(D)** Differential abundance analysis: left panel shows linear discriminant analysis (LDA) scores highlighting microbial taxa differences between DSS and Kimate-X + Kimate-F groups. Right panel shows relative abundance changes. Data are presented as mean ± SEM, statistical significance is indicated by *p < 0.05, **p < 0.01, and ***p < 0.001.

In the metagenomic analysis of canine fecal samples, microbial diversity and community composition were compared between the DSS group and the Kimate-X + Kimate-F group. The Shannon diversity index revealed significantly higher microbial diversity in the Kimate-X + Kimate-F group compared to the DSS group (Figure 4A, *p* < 0.05), although the InvSimpson index did not show a significant difference (*p* > 0.05). Principal coordinate analysis (PCoA) (Figure 4B, left) demonstrated partial separation of microbial community structures between the two groups, indicating structural differences. However, Bray–Curtis distance analysis (Figure 4B, right) did not show statistically significant differences between the two groups (*p* > 0.05).

Microbial abundance analysis (Figure 4C) revealed distinct microbial compositions at the phylum, family, genus, and species levels in the Kimate-X + Kimate-F group compared to the DSS group. Specifically, the Kimate-X + Kimate-F group exhibited a higher relative abundance of Firmicutes, with significant increases in *Bifidobacterium*. LEfSe analysis (Figure 4D) further highlighted that *Limosilactobacillus oris* was significantly enriched in the Kimate-X + Kimate-F group, whereas *Tyzzerella nexilis*, *Coprococcus comes*, and *Prevotella copri* clade A were more abundant in the DSS group.

### Canine gut metabolic pathway analysis

**Figure 5:**
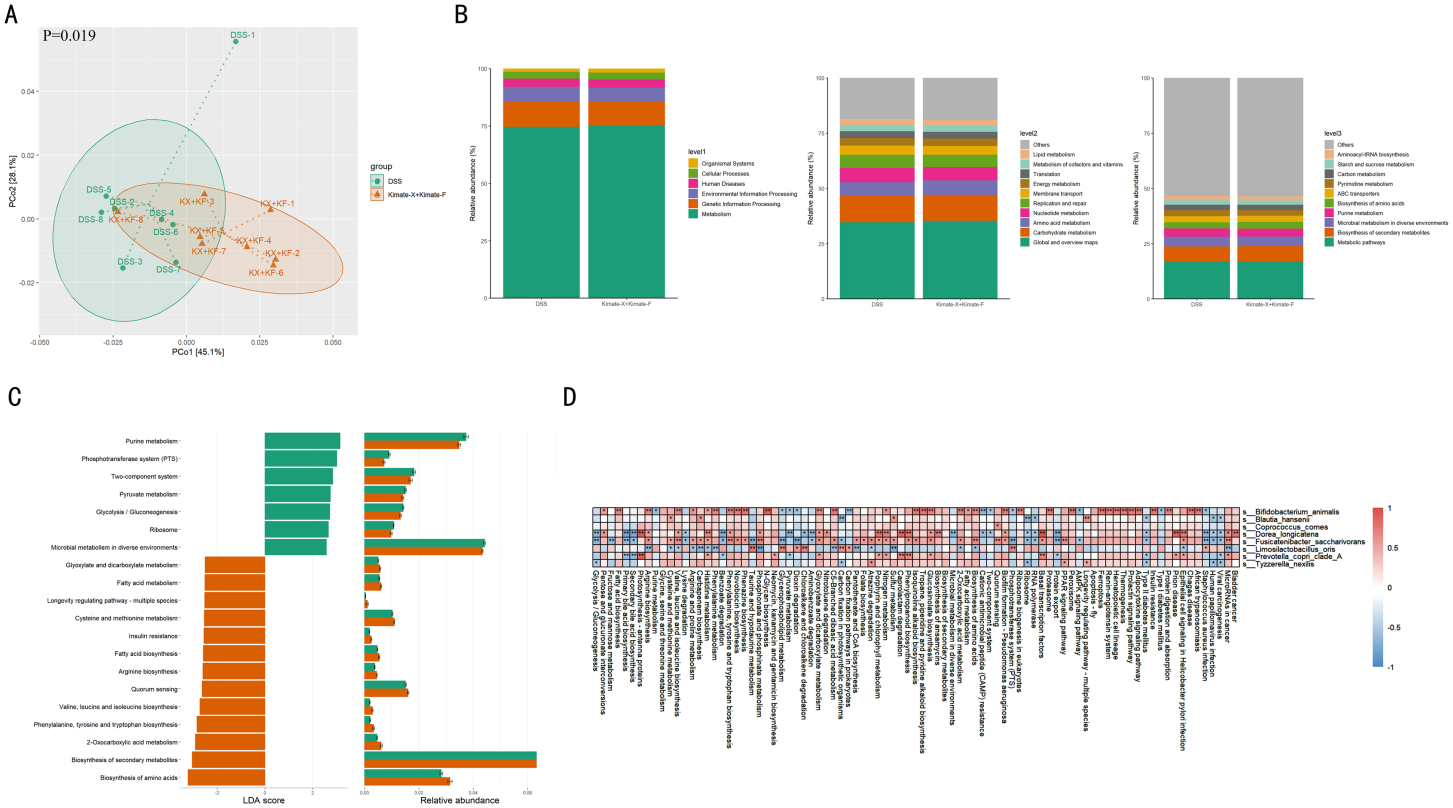
Effects of Kimate-X and Kimate-F on metabolic pathways in DSS-induced canine colitis model. **(A)** PCoA analysis of metabolic pathway data, illustrating separation between DSS and Kimate-X + Kimate-F groups. Ellipses represent 95% confidence intervals. **(B)** Functional prediction (KEGG database): primary metabolic function classification (left), secondary (middle), and tertiary (right) functions with their relative abundances. **(C)** LEfSe analysis: LDA scores (left) and relative abundance changes (right) for differential metabolic pathways. **(D)** Correlation heatmap: associations between key microbes and metabolic pathways. Red indicates a positive correlation, blue indicates a negative correlation, and the deeper the color, the stronger the correlation. Data are presented as mean ± SEM, statistical significance is indicated by *p < 0.05, **p < 0.01, and ***p < 0.001.

Comparing gut metabolic pathways between the DSS and Kimate-X + Kimate-F groups revealed significant differences. PCoA results (Figure 5A) showed distinct metabolic pathway compositions between the two groups (*p* = 0.019), indicating differences in metabolic pathway diversity and composition. Figure 5B displays functional pathway differences across various levels, with the Kimate-X + Kimate-F group showing higher relative abundances in pathways related to metabolism, environmental information processing, and cellular processes, particularly key metabolic pathways such as amino acid, carbohydrate, and lipid metabolism. LDA analysis (Figure 5C) revealed significant differences in specific metabolic pathways. The DSS group was enriched in pathways such as purine metabolism, the phosphotransferase system (PTS), glycolysis/gluconeogenesis, and pyruvate metabolism, while the Kimate-X + Kimate-F group exhibited higher enrichment in fatty acid metabolism, amino acid biosynthesis, secondary metabolite biosynthesis, cysteine and methionine metabolism, and longevity regulation pathways.

Figure 5D illustrates the correlations between differential gut microbiota and various metabolic pathways in DSS-induced canine colitis. Several bacterial species show significant positive or negative correlations with specific metabolic pathways. *Bifidobacterium animalis* demonstrates positive correlations with carbohydrate metabolism, specifically pentose and glucuronate interconversions (*p* < 0.05), fatty acid biosynthesis, arginine biosynthesis, and several amino acid biosynthesis pathways, including those for valine, leucine, and isoleucine, as well as phenylalanine, tyrosine, and tryptophan (*p* < 0.05). It is also positively correlated with the PPAR signaling pathway while being negatively correlated with the AMPK signaling pathway (*p* < 0.05). *Blautia hansenii* is positively correlated with cysteine and methionine metabolism (*p* < 0.05), and with the PPAR signaling pathway, but shows a negative correlation with the AMPK signaling pathway. *Coprococcus comes* is similarly positively correlated with the PPAR signaling pathway and negatively correlated with the AMPK signaling pathway. *Dorea longicatena* is primarily involved in amino acid synthesis and degradation, showing positive correlations with arginine biosynthesis, valine, leucine, and isoleucine biosynthesis, phenylalanine, tyrosine, and tryptophan biosynthesis (*p* < 0.05), and histidine metabolism (*p* < 0.01), while being negatively correlated with lysine degradation (*p* < 0.05). In terms of inflammatory pathways, it also exhibits a positive correlation with the PPAR signaling pathway and a negative correlation with the AMPK signaling pathway. *Fusicatenibacter saccharivoran* shows positive correlations with arginine biosynthesis, valine, leucine, and isoleucine biosynthesis, and phenylalanine, tyrosine, and tryptophan biosynthesis (*p* < 0.05), alongside other amino acid metabolic pathways like cysteine and methionine metabolism, and arginine and proline metabolism (*p* < 0.05). It is negatively correlated with lysine degradation (*p* < 0.05), positively correlated with the PPAR signaling pathway (*p* < 0.01), and negatively correlated with the AMPK signaling pathway (*p* < 0.05). *Limosilactobacillus oris* shows negative correlations with the biosynthesis of several amino acids, including phenylalanine, tyrosine, and tryptophan, and arginine biosynthesis (*p* < 0.01), as well as valine, leucine, and isoleucine biosynthesis (*p* < 0.05). It also negatively correlates with amino acid metabolic pathways such as cysteine and methionine metabolism, histidine metabolism, and C5-branched dibasic acid metabolism (*p* < 0.05). Additionally, it is negatively correlated with the PPAR signaling pathway and positively correlated with the AMPK signaling pathway. *Prevotella copri* clade A shows positive correlations with histidine metabolism (*p* < 0.05), and with the PPAR signaling pathway, while being negatively correlated with the AMPK signaling pathway. Finally, *Tyzzerella nexilis* is positively correlated with the PPAR signaling pathway (*p* < 0.05) and negatively correlated with the AMPK signaling pathway.

### Canine fecal short-chain fatty acids analysis

**Figure 6:**
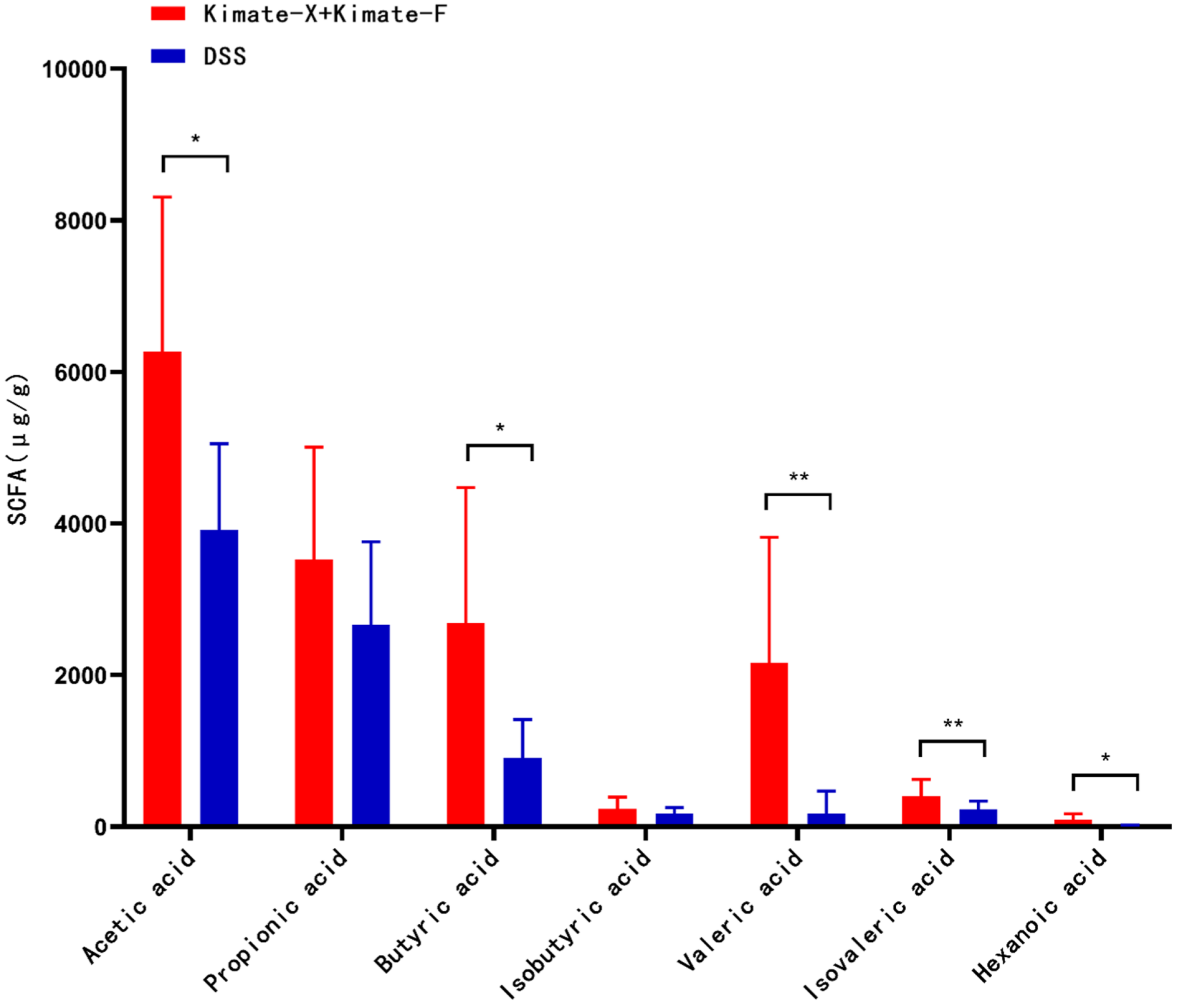
Effects of Kimate-X and Kimate-F on short-chain fatty acids (SCFAs) in DSS-induced canine colitis model. Relative abundances of major SCFAs (acetate, propionate, butyrate, pentanoate, isovalerate, and hexanoate) in feces from Kimate-X + Kimate-F and DSS groups. Data are presented as mean ± SEM, statistical significance is indicated by *p < 0.05, **p < 0.01, and ***p < 0.001.

In the comparison of SCFAs between the two groups of dogs (Figure 6), the Kimate-X + Kimate-F group showed significantly higher levels of acetic acid (*p* < 0.05), butyric acid (*p* < 0.05), valeric acid (*p* < 0.01), isovaleric acid (*p <* 0.01), and hexanoic acid (*p* < 0.05) compared to the DSS group. This indicates that the Kimate-X + Kimate-F group produced higher overall amounts of SCFAs. Although no significant differences were observed in propionic and butyric acid levels between the two groups, the Kimate-X + Kimate-F group exhibited higher overall SCFAs concentrations.

### Correlation between fecal short-chain fatty acids, pro-inflammatory factors, and microbiota

**Figure 7:**
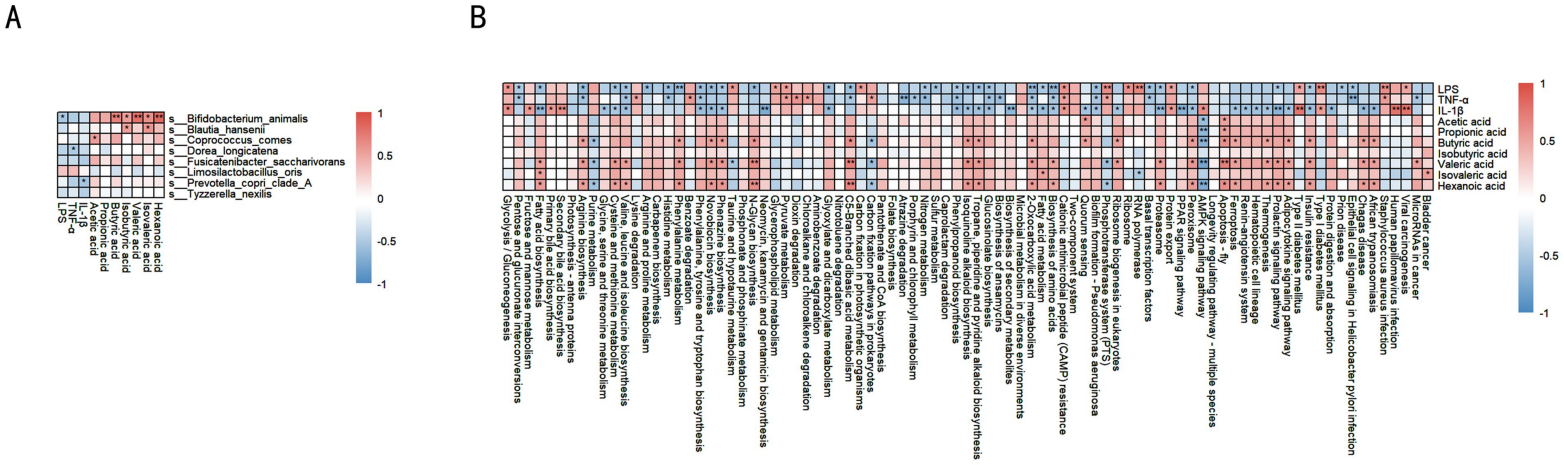
Correlation analysis between key microbes, SCFAs, and inflammatory factors. **(A)** Correlation heatmap: relationships between different microbes and SCFAs, along with inflammatory factors (LPS, TNF-α, IL-1β). Red indicates positive correlation, blue indicates negative correlation. **(B)** Correlation heatmap: associations between different metabolic pathways, SCFAs, and inflammatory factors. Red indicates a positive correlation, blue indicates a negative correlation, and the deeper the color, the stronger the correlation. Data are presented as correlation coefficients, statistical significance is indicated by *p < 0.05, **p < 0.01, and ***p < 0.001.

In the DSS-induced colitis model in dogs, significant correlations were observed between specific microbiota and SCFAs (e.g., acetic acid, propionic acid, butyric acid, isovaleric acid, valeric acid, hexanoic acid), as well as pro-inflammatory markers (LPS, TNF-α, IL-1β) (Figure 7A). For example, *Bifidobacterium animalis* showed significant positive correlations with isovaleric acid and hexanoic acid (*p* < 0.01), and negative correlations with LPS (*p* < 0.05). *Blautia hansenii* was positively correlated with isovaleric acid, valeric acid, LPS (*p* < 0.05), and TNF-α (*p* < 0.01). *Coprococcus comes* was positively correlated with isovaleric acid, valeric acid (*p* < 0.05), and LPS (*p* < 0.05). *Dorea longicatena* showed significant positive correlations with isovaleric acid (*p* < 0.05) and hexanoic acid (*p* < 0.01). Both *Fusicatenibacter saccharivorans* and *Limosilactobacillus oris* showed significant positive correlations with hexanoic acid (*p* < 0.05). *Prevotella copri* clade A showed significant positive correlations with isovaleric acid (*p* < 0.01) and LPS (*p* < 0.05). *Tyzzerella nexilis* was positively correlated with valeric acid (*p* < 0.01) and isovaleric acid (*p* < 0.01).

### Correlation between fecal short-chain fatty acids, pro-inflammatory factors, and metabolic pathways

In the DSS-induced colitis model in dogs, SCFAs are associated with various metabolic pathways (Figure 7B). Acetate is positively correlated with branched-chain amino acid synthesis, phenylalanine, tyrosine, and tryptophan biosynthesis, fatty acid metabolism, fatty acid degradation, and glycolysis/gluconeogenesis. Propionate shows positive correlations with branched-chain amino acid metabolism and fatty acid metabolism. Butyrate is positively correlated with arginine biosynthesis and phenylalanine metabolism (*p* < 0.05), and also with branched-chain amino acid synthesis and fatty acid synthesis pathways. Isovalerate is positively correlated with phenylalanine, tyrosine, and tryptophan biosynthesis, phenylalanine metabolism, branched-chain amino acid synthesis, and fatty acid metabolism (*p* < 0.05). Pentanoate is positively correlated with fatty acid synthesis (*p* < 0.05). Hexanoate shows significant positive correlations with fatty acid metabolism and amino acid metabolism, including arginine, proline, cysteine, and methionine metabolism, as well as branched-chain amino acid synthesis (*p* < 0.05).

Regarding pro-inflammatory markers, LPS is negatively correlated with fatty acid metabolism, cysteine and methionine metabolism, histidine metabolism, and arginine biosynthesis (*p* < 0.05). TNF-α is negatively correlated with fatty acid metabolism, glycolysis/gluconeogenesis, and several amino acid metabolic pathways (*p* < 0.05). Similarly, IL-1β is negatively correlated with fatty acid synthesis and multiple amino acid metabolic pathways (*p* < 0.05).

## Discussion

The study explores the mechanisms by which probiotics *Enterococcus faecium* Kimate-X and *Lactobacillus plantarum* Kimate-F, along with their combination, alleviate canine inflammatory bowel disease. By employing a comprehensive evaluation that includes *in vitro* antibacterial assays, cell models, mouse models, and canine models, we systematically assessed the anti-inflammatory effects of these probiotics and elucidated their regulatory roles in gut microbiota and metabolic pathways.

In the *in vitro* antibacterial assays, Kimate-F demonstrated significantly stronger antibacterial activity against *Escherichia coli*, *Salmonella Enteritidis*, and *Yersinia enterocolitica* compared to Kimate-X. This may be attributed to the ability of *Lactobacillus plantarum* to produce higher levels of organic acids, hydrogen peroxide, and bacteriocins—potent antimicrobial substances (43). Organic acids reduce local pH, inhibiting pathogenic bacteria growth, while hydrogen peroxide and bacteriocins disrupt bacterial cell membranes, causing lysis (44). In contrast, Kimate-X exhibited relatively weaker antibacterial activity, likely due to its limited production of antimicrobial substances. However, Kimate-X demonstrated more pronounced effects in regulating immune responses.

In the LPS-induced RAW 264.7 macrophage inflammation model, Kimate-X significantly reduced NO and TNF-α production, outperforming Kimate-F. This suggests that Kimate-X holds unique advantages in immune regulation. Previous studies have indicated that *Enterococcus faecium* can modulate Toll-like receptor (TLR) signaling pathways through its cell wall components, such as lipoteichoic acid (LTA) and peptidoglycan, thereby inhibiting NF-κB activation and reducing pro-inflammatory cytokine secretion (45, 46). Additionally, *Enterococcus faecium* may enhance immune tolerance by inducing the production of anti-inflammatory cytokines such as IL-10.

To further investigate the probiotic functions of Kimate-X and Kimate-F, we conducted experiments using a DSS-induced colitis model in mice. Both Kimate-X, Kimate-F, and their combination significantly ameliorated symptoms of inflammation, including weight loss, DAI, elevated spleen index, and shortened colon length. Histopathological analysis revealed that intestinal mucosal damage in the combination group was significantly less than in the single-strain groups, indicating a synergistic effect between the probiotics. The combination group also showed significantly greater reductions in serum LPS and TNF-α levels, alongside an increase in IL-10 levels, compared to the single-strain groups. This synergy may arise from complementary mechanisms of the two probiotics in regulating gut microbiota, barrier function, and immune responses.

Based on the enhanced probiotic potential observed in the combination group from *in vitro* and mouse experiments, we selected the combination of Kimate-X and Kimate-F for further experimentation. In the DSS-induced colitis model in dogs, the combination group significantly improved weight gain, mental status, and fecal characteristics, while markedly reducing serum levels of Pro-inflammatory factors (LPS, TNF-α, IL-1β), Significantly increased the levels of the anti-inflammatory factor IL-10. These results further confirm the efficacy of probiotics in large animal models, bolstering their potential for clinical application.

To explore the mechanisms underlying the effects of the Kimate-X and Kimate-F combination in alleviating canine colitis, we performed metagenomic analysis on fecal samples from both groups. The results indicated significantly higher gut microbial diversity in the combination group compared to the DSS group, with increased relative abundance of *Firmicutes*. Notably, the enrichment of *Bifidobacterium* species may contribute to inhibiting pathogen colonization and enhancing gut barrier function (47, 48).

LEfSe analysis further highlighted significant differences in characteristic microbes between the two groups. The Kimate-X + Kimate-F combination not only significantly improved the composition and structure of the canine gut microbiota but also affected host metabolic functions. Comparative analysis of metabolic pathways revealed notable differences between the groups, indicating that the combination intervention effectively altered the gut metabolic profile. The Kimate-X + Kimate-F group exhibited higher relative abundances in gene pathways related to metabolism, environmental information processing, and cellular processes, particularly in key metabolic pathways such as amino acid metabolism, carbohydrate metabolism, and lipid metabolism. These pathways are closely linked to energy supply, nutrient absorption, and immune regulation, suggesting that Kimate-X + Kimate-F may promote overall health and metabolic function in dogs by enhancing these pathways (49, 50).

LDA analysis further revealed differences in specific metabolic pathways between the groups. The DSS group was enriched in pathways such as purine metabolism, the phosphotransferase system (PTS), glycolysis/gluconeogenesis, and pyruvate metabolism, findings that align with previous studies on DSS-induced colitis (51, 52). The upregulation of these pathways may be associated with inflammatory responses and metabolic imbalances (53–55). In contrast, the Kimate-X + Kimate-F group was enriched in pathways related to fatty acid metabolism, amino acid biosynthesis, secondary metabolite biosynthesis, cysteine and methionine metabolism, and longevity regulation pathways. Activation of these pathways may contribute to antioxidant, anti-inflammatory, and cell-protective effects, potentially delaying aging and enhancing immune function (56, 57). Thus, Kimate-X + Kimate-F supplementation may improve canine gut health by regulating gut microbiota metabolic functions, promoting beneficial metabolite production, and inhibiting inflammation-related pathways.

SCFAs, which are products of gut microbial fermentation, play essential roles in regulating immunity and maintaining gut barrier integrity (58, 59). Many gastrointestinal diseases in dogs, such as inflammatory bowel disease and diarrhea, are associated with dysbiosis of the gut microbiota and alterations in metabolic by-products. Among these, changes in SCFA levels and the microbial populations responsible for their production are key characteristics (60, 61). Given the enrichment of fatty acid metabolism pathways in the Kimate-X + Kimate-F group, we compared fecal SCFA levels between groups. The combination group exhibited significantly higher levels of SCFAs, such as acetic acid, butyric acid, pentanoic acid, isovaleric acid, and hexanoic acid. By increasing SCFA levels, the combination group demonstrated its potential to promote gut health and metabolic regulation.

In the analysis of the correlation between differential species and metabolic pathways. We observed a significant enrichment of *Limosilactobacillus oris* in the DSS group, which exhibited a negative correlation with the PPAR signaling pathway and a positive correlation with the AMPK signaling pathway. This negative correlation suggests that *Limosilactobacillus oris* may inhibit lipid metabolism and suppress anti-inflammatory responses, while its positive correlation with AMPK implies a propensity to promote catabolic activity and energy production. This regulatory pattern contrasts sharply with the microbiota in the probiotic group, where *Bifidobacterium animalis*, *Dorea longicatena*, and *Fusicatenibacter saccharivorans* demonstrated positive correlations with PPAR signaling and negative correlations with AMPK signaling. These findings suggest that these strains tend to enhance anabolic metabolism and anti-inflammatory responses, thus contributing to intestinal repair and energy homeostasis.

The complex interaction between microbial metabolism and inflammatory pathways in inflammatory bowel disease is well-established (62). Our study further revealed correlations between specific microbes, SCFAs, and pro-inflammatory factors. *Bifidobacterium animalis* was positively correlated with isovaleric acid and hexanoic acid while being negatively correlated with LPS, suggesting that Kimate-X + Kimate-F supplementation may exert anti-inflammatory effects by increasing SCFA production (63, 64). Similarly, *Blautia hansenii* showed positive correlations with isovaleric acid, pentanoic acid, LPS, and TNF-α, indicating a role in inflammation regulation via SCFA metabolism (65). The positive correlations of *Coprococcus comes* and *Dorea longicatena* with isovaleric acid and hexanoic acid reinforce the pivotal role of SCFAs in regulating inflammation. Additionally, *Prevotella copri* clade A was positively correlated with isovaleric acid and LPS, suggesting that some microbes may have dual roles in SCFA production and pro-inflammatory responses (66). These findings imply that specific microbial groups regulate gut inflammation by modulating SCFA metabolism and impacting the expression of pro-inflammatory factors. The observed correlations between LPS, TNF-α, and IL-1β with key metabolic pathways suggest that pro-inflammatory factors exacerbate inflammation by disrupting metabolic balance, further weakening gut barrier function and triggering immune responses (67). These insights into the microbiota-metabolism-inflammation axis provide potential therapeutic targets for managing canine inflammatory bowel disease through probiotic or microbial interventions.

Additionally, previous research on canine probiotics has highlighted that factors such as administered dose, feeding duration, and specific formulation can significantly influence therapeutic outcomes (68, 69). In our study, we pre-fed the dogs with a combined formula of Kimate-X and Kimate-F for 1 week before inducing colitis, during which no adverse symptoms or abnormal clinical signs were observed. This observation underscores the safety profile of these probiotics in canine models. Nonetheless, further research is warranted to systematically evaluate varying doses, extend feeding durations, and explore different formulations to optimize the benefits of probiotic therapy for canine IBD.

Moreover, safety is a critical consideration in clinical applications of probiotics. Before DSS induction, we performed a one-week regimen of probiotic feeding, during which the dogs did not exhibit any abnormalities, providing preliminary evidence that the administered strains do not elicit adverse effects in dogs. In summary, this study systematically demonstrated that the combination of *Enterococcus faecium* Kimate-X and *Lactobacillus plantarum* Kimate-F alleviates canine IBD through multiple mechanisms, including direct pathogen inhibition, immune modulation, increased SCFA production, and regulation of gut microbiota and metabolic pathways. These findings provide strong scientific support for the clinical application of probiotics in improving canine gut health and treating IBD.

## Data Availability

The sequencing data generated in this study have been submitted to the NCBI BioSample database under accession numbers SAMN48004212–SAMN48004227.
